# Cancer genetic testing uptake in the primary care setting: Patient perspectives on barriers and facilitators throughout the testing process

**DOI:** 10.1002/jgc4.70195

**Published:** 2026-03-26

**Authors:** Tesla N. Theoryn, Faith Beers, Emerson J. Dusic, Catharine Wang, DaLaina Cameron, Heather Harris, Sarah Knerr, Elizabeth M. Swisher, Susan Brown Trinidad

**Affiliations:** ^1^ University of Washington Seattle Washington USA; ^2^ Boston University School of Public Health Boston Massachusetts USA

**Keywords:** genetic testing, hereditary cancer, provider communication

## Abstract

Genetic testing for gene variants associated with hereditary cancers can help with cancer prevention, early detection, and treatment. However, testing has not been well integrated into primary care settings where its preventative impact can be realized. To explore patient‐level barriers and facilitators throughout the genetic testing process in primary care settings, we conducted a thematic analysis of semi‐structured interviews with 31 patients within the Early Detection of Genetic Risk (EDGE) study who had not completed the risk assessment (*n* = 2), had completed the risk assessment but were ineligible (*n* = 8), had declined testing (*n* = 10), and had completed testing (*n* = 11). Interviewees were broadly interested in genetic testing. Those who did not complete the risk assessment cited limited access to technology, exacerbated by health and financial struggles. Several interviewees who completed the risk assessment but were deemed ineligible for testing indicated that their lack of knowledge about biological relatives prevented complete responses to the risk assessment. Those who opted out of testing cited concerns over privacy, insurance discrimination, and potential psychological burden. Notably, the majority who declined testing were unsure if they would refuse again in the future, and three went on to request genetic testing after being invited to complete an interview. Those who changed their minds about testing stated changes in life circumstances (such as obtaining life insurance) that facilitated openness to testing. Patients who completed testing shared similar concerns to those who declined but were motivated by their familial cancer history and believed genetic testing could lead to preventative options. A key finding of this study was that patient readiness for testing changed over relatively brief follow‐up times. These results highlight the need for practicable approaches to re‐offering genetic testing to individuals over time.

## 1 INTRODUCTION

For over 20 years, cancer has been a leading cause of morbidity and mortality in the United States (CDC, 2023), with inherited variants associated with 5%–10% of cases (Garber & Offit, 2005). Proactive identification of individuals who carry such variants is critical to reducing hereditary cancer burden. Genetic testing for hereditary cancer risk (GT) can facilitate interventions aimed at early detection and prevention, such as enhanced screenings and risk‐reducing surgeries (Cragun et al., 2020). The reach of early identification can be increased by extending GT from primarily cancer care settings to medical spaces focused on prevention, such as primary care (Chambers et al., 2015; Dusic et al., 2022; Petry et al., 2019). In the primary care setting, for example, providers can identify patients who may benefit from GT based on family history, order testing, and align cancer screenings according to clinical recommendations based on GT results. Ultimately, this integration would facilitate early detection of hereditary cancers.

Efforts to incorporate GT into primary care have been underway for over two decades, and clinical testing recommendations have been written accordingly (Bednar et al., 2022; Dieng et al., 2014; McCuaig et al., 2018; US Preventive Services Task Force et al., 2019). However, many people who may benefit are still not being tested (Childers et al., 2018). Primary care providers (PCPs) are best positioned to address this gap, but they face significant systemic challenges, including limited opportunities for genetics education and access to genetic specialists, incomplete or non‐intuitive integration of genetic information within the electronic health record (EHR), and competing clinical priorities paired with short patient visits (Dusic et al., 2022; Evenson et al., 2016; Harding et al., 2019). Added to this, patient circumstances and concerns mean that when a provider is ultimately able to offer GT, the patient may not be situated to move forward with it (Actkins et al., 2021; Keogh et al., 2017). Recognizing that patient–provider interaction is still critical to facilitating GT, there is a need to identify feasible provider strategies, informed by patient experiences, to improve the delivery of genetic testing services.

A substantial body of work is dedicated to understanding why people who may benefit from testing do not pursue it. Systematic reviews cataloging the barriers have captured perspectives from a range of populations, including those with a personal history of cancer, those with a family history of cancer, and the general population (including participants from both specialty and primary care settings) (Keogh et al., 2017; Shen et al., 2022; Smith‐Uffen et al., 2021). Concerns over perceived cost, psychological harm, clinical utility, and privacy were consistently identified as key barriers. However, the earliest barriers to engagement with the testing process are not well captured, as those who are not eligible for testing, or who do not start the process, are often excluded from studies. Further, barriers are often looked at in aggregate rather than at specific stages in the testing process. The current approach to genetic testing for hereditary cancer consists of not one, but multiple steps toward completion, each of which represents an opportunity for patients to drop out of or be removed from the testing process (Bednar et al., 2022; Dusic et al., 2022). How barriers and considerations change as the GT process progresses is poorly understood, which limits our capacity to develop evidence‐based interventions. One study with patients with breast cancer did find a difference in why patients declined testing based on when they dropped out of the process – those who declined immediately saw little relevance to testing, while those who declined later had more psychological reasons (Schlich‐Bakker et al., 2007). In line with the idea that barriers and considerations change throughout the testing process, we are also looking to explore how they continue to change following initial dropout. Only one identified study has explicitly focused on decision permanence (Halverson et al., 2020). The study, conducted by Halverson and colleagues at a hereditary cancer clinic, found that the majority (67%) of individuals who declined testing would change their minds if their clinician offered it again. The literature on these topics in cancer care settings is sparse, and how they are reflected in the primary care setting, where the function of GT is slightly different, is entirely unknown.

A more complete understanding of the nature of patient barriers and thoughts around GT can inform provider communication, suggesting potential interventions and improvements to increase uptake of testing in the context of hereditary cancers and primary care. As such, we conducted a qualitative study to answer the question of how considerations change across the GT process and what is the permanence of these decisions among primary care patients.

## 2 METHODOLOGY

### 2.1 Parent study and participant population

The Early Detection of Genetic Risk (EDGE) study was a cluster‐randomized controlled trial that evaluated two engagement strategies for completing cancer risk assessments: direct‐to‐patient (completed independently online) and point‐of‐care (in clinic or over the phone). Regardless of arm, the risk assessment was offered prior to a patient's upcoming appointment, so as to allow participants the opportunity to discuss the study and GT with their provider. Providers were offered an optional training at the start of the study for continued medical education credit, focused on the process of identifying and following‐up with high‐risk patients (Swisher et al., 2025). The study was conducted across two large healthcare systems with clinics in Washington, Wyoming, and Montana (Swisher et al., 2025).

After completing the cancer risk assessment online or in person, everyone was treated the same in both arms. Those with a sufficient family or personal history of cancer were deemed eligible for at‐home GT (Bowen et al., 2021; Swisher et al., 2025). All test‐eligible patients were sent an overview of the next steps and a link to the study website with frequently asked questions (FAQs) about genetic testing (Appendix S1). The FAQs went over potential impact on health insurance, utility of testing, genes tested, data privacy, and the testing process. However, no pre‐test counseling was provided, where this information, along with alternatives to testing and implications for risk, would have likely been reviewed in more depth. For all eligible patients, one follow‐up phone call was made by study staff to explain the logistics of the process, answer questions according to the study FAQs, and offer/order the at‐home GT through Color Health (www.color.com) at no cost. If a participant was interested in moving forward with testing, a provider placed the order for the testing kit. Kits were sent to the participant's provided address. To complete testing, participants provided a saliva sample, mailed the testing kit back to Color Health, and set up an online account to access their results. Results were uploaded to participants' EHR and were available via the participant's Color online account. Genetic counseling was offered for all individuals for whom a pathogenic variant was identified. Participants were given options to opt out of GT or the study at large (interviews and surveys) at each contact point. Main outcomes of the EDGE study, along with the protocol, were published previously (Bowen et al., 2021; Swisher et al., 2025).

**FIGURE 1:**
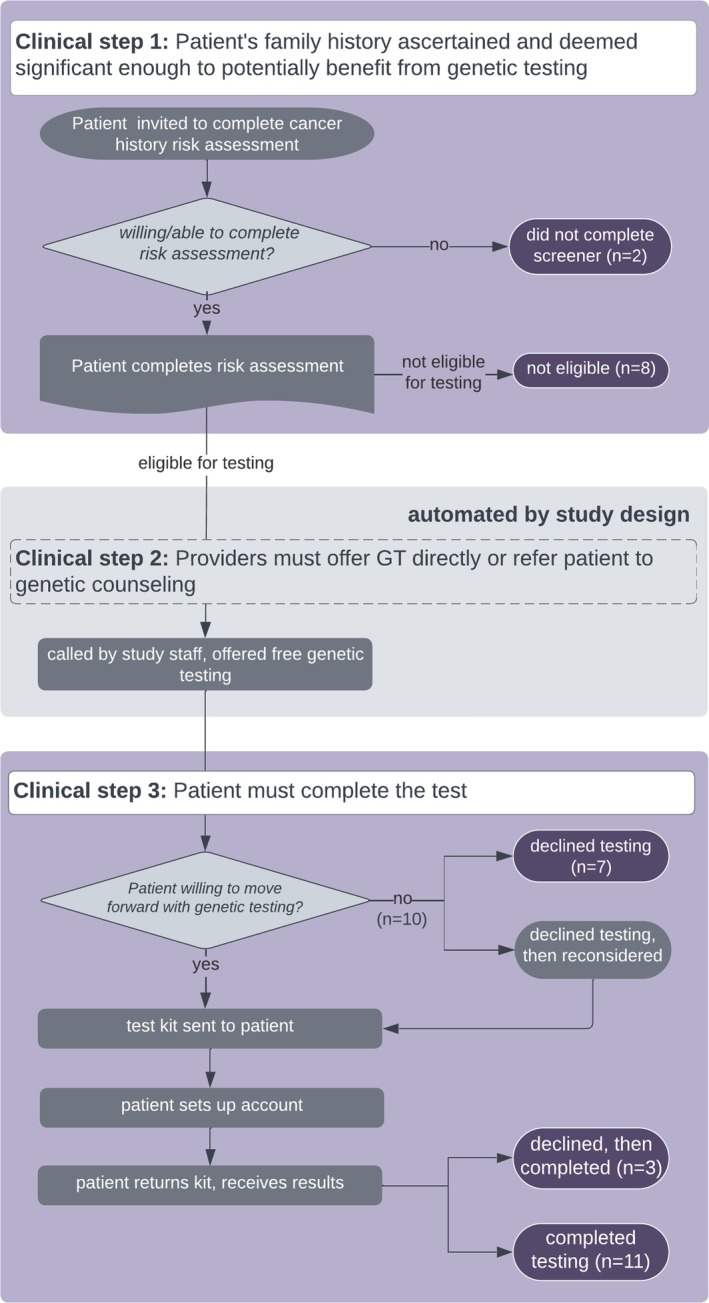
Primary care testing model and interview groups.

EDGE invited more than 59,500 primary care patients to complete the cancer history risk assessment. Of the 13,705 individuals who completed the risk assessment, GT was offered to the 4673 who met cancer history criteria. Of these, 1,474 (31.5%) completed GT. We conducted semi‐structured interviews (*N* = 31) with participants at four stages in this GT process, those who: did not complete the initial risk assessment (*n* = 2), completed the assessment but were ineligible due to an insufficient personal or familial history of cancer (*n* = 8), were eligible and declined (*n* = 10), and completed GT (*n* = 11) (Figure 1). Participant quotes are labeled according to their stage of drop‐out. ‘Early’ if they did not complete the risk assessment or were ineligible due to insufficient cancer history, ‘Late’ if they opted out of testing following eligibility, ‘Switched’ if they initially declined but later requested testing, and ‘Completed’ if they completed testing. This study was approved by the University of Washington Institutional Review Board (STUDY00009476) and is reported using guidance from the Reflexive Thematic Analysis Reporting Guidelines (RTARG) (Braun & Clarke, 2024).

### 2.2 Recruitment

EDGE study participants were English‐speaking individuals aged 25 years and older with scheduled appointments at a participating clinic. Individuals were ineligible for this nested qualitative study if they stated they did not want to be contacted for future research. For the interviews, we employed a purposive sampling strategy to select study participants who varied in gender, age, study arm, and clinic system. We aimed to recruit 40 individuals from the EDGE study to gather general perspectives and thoughts about genetic testing and sampled specifically for participants who dropped out at distinct stages of the testing process to capture unique considerations.

Potential interviewees were contacted via email up to three times. Emails were sent by the interviewer (TNT), who introduced herself, stated the purpose of the study, and provided a brief overview of the topics. Because of challenges in recruiting individuals who did not complete the risk assessment, those who called in to express that they were not moving forward with it were also invited to interview. Participant contact data was stored in the UW's HIPAA‐compliant REDCap system.

### 2.3 Data generation

Our research question focuses on why people drop out at different stages of the GT process, and the permanence of their decision. To answer this exploratory, subjective question, we utilize qualitative methods for data generation and reflexive thematic analysis (RTA) for interpretation. These methods provide the flexibility to offer insight into the many complex paths and perspectives of individuals moving through GT. Semi‐structured interview guides were developed based on the stage of drop‐out and pilot‐tested with study staff, with only minor wording and question order changes. Topics included: general thoughts about GT, understanding of GT in the context of the study, reasons for considering GT, concerns or barriers, interactions with family members and physicians, and the permanence of their decision. Demographic information was also collected (Appendix S1).

A research assistant (TNT) with formal training as an educator and an interest in GT and implementation work completed: patient follow‐up after the risk assessment, participant recruitment for interviews, the one‐on‐one interviews, and led the analysis for this study. She was trained under a qualitative research expert (SBT). Recruitment and interviews took place between 2022 and 2023. The interviews were conducted on Zoom, with participants calling or videoconferencing. The interviews averaged approximately 22 min (range: 9–60 min), and participants were offered a $10 gift card for their time. The sessions were digitally recorded (audio and video), transcribed with filler words removed for clarity in line with our pragmatic paradigmatic approach, and de‐identified prior to analysis.

### 2.4 Data analysis

The codebook was initially developed with deductive codes derived from the interview guide. Initial code groups and associated example codes include genetic testing (opinions on testing, reaction, etc.), considerations (research, cost, discrimination, etc.), feedback (time, questions, etc.), and misc. (other testing, etc.). While the use of a starting codebook allowed for alignment with our research question, we also reviewed the transcripts to allow for inductive code generation more in line with RTA. Further, codes were continuously refined throughout the coding process. EJD and TNT used ATLAS.ti software to code the transcripts. A random sample of three transcripts was selected and double‐coded at various stages in the coding process (beginning, middle, and end). The coders met with a senior qualitative expert (SBT) to discuss the code choices and assumptions, aiming for a more in‐depth understanding of the data and codes, focusing on areas where there were coding discrepancies and updating or adding codes when needed to ensure the final codes captured the coders' interpretations. The remaining transcripts were then divided and coded individually by the two coders, with the analysts serving as sounding boards throughout the process.

We (TNT, FB, EJD, SBT) performed a RTA, framed by pragmatism as a research paradigm, allowing us to focus on the practical consequences of our findings (Braun & Clarke, 2021a, 2021b; Wainstein et al., 2023). This involved recursively conducting six phases of analysis: data familiarization, coding, identifying initial themes, reviewing themes, refining themes, and writing up the findings. Barriers and facilitators were broken down based on when participants dropped out of, or were removed from, the testing process, focusing on clinically relevant stages: early (did not complete cancer history risk assessment or completed the assessment and were ineligible) and late drop‐out (test‐eligible individuals who declined GT). The study arm was considered only for the earliest stage of drop‐out, as all points following completion of the risk assessment were identical in the study procedure and staff contact.

## 3 ANALYSIS

### 3.1 Response rate and participant demographics

The interview response rate varied based on the testing process stage. Of those approached for interviews, those who did not complete the risk assessment or declined GT had the lowest response rates (2/49, 4.1% and 10/174, 5.5%, respectively). Those who were ineligible for GT due to insufficient personal or familial cancer history had the highest response rate (8/17, 47.1%), followed by those who completed GT (11/62, 17.8%). Thematic sufficiency was met overall around the motivations for GT and for the barriers following the risk assessment. However, themes relevant specifically to the subset of individuals who did not complete the initial risk assessment (i.e., did not engage in the study at all) did not reach sufficiency. For this group, recruitment efforts were largely unsuccessful. However, two individuals reached out to the study staff and were invited to participate at that time. These interviews were included to represent the reported experience, but there are no claims that they represent a comprehensive view of the earliest barriers to testing.

Interview participants predominantly identified as white (88%) and female (60%). The median age was 58 years old and participants were roughly evenly split between healthcare systems (Table 1). Of the two individuals who reported a personal history of an assessed hereditary cancer, both completed GT.

**TABLE 1:** Participant demographics.

Demographics	Interview groups					Total (*n* = 31)
	Did not complete risk assessment (*n* = 2)	Ineligible for GT (*n* = 8)	Declined GT (*n* = 7)	Declined, then completed GT (*n* = 3)	Completed GT (*n* = 11)	
Sex						
Male	1 (50%)	5 (62.5%)	3 (42.9%)	1 (33.3%)	2 (18.2%)	12 (38.7%)
Female	1 (50%)	3 (37.5%)	4 (57.1%)	2 (66.7%)	9 (81.8%)	19 (61.3%)
Race						
White	2 (100%)	4 (50%)	7 (100%)	3 (100%)	11 (100%)	27 (87.1%)
Asian	–	4 (50%)	–	–		4 (12.9%)
Ethnicity						
Not Hispanic or Latino	2 (100%)	7 (87.5%)	6 (85.7%)	3 (100%)	10 (90.9%)	28 (90.3%)
Hispanic or Latino	–	1 (12.5%)	1 (14.3%)	–	1 (9.1%)	3 (9.7%)
Age						
25–45	–	4 (50%)	2 (28.6%)	1 (33.3%)	5 (45.5%)	12 (38.7%)
46–65	1 (50%)	3 (37.5%)	2 (28.6%)	1 (33.3%)	2 (18.2%)	9 (29.0%)
>65	1 (50%)	1 (12.5%)	3 (42.9%)	1 (33.3%)	4 (36.4%)	10 (32.3%)
Clinic system state						
Washington	1 (50%)	5 (62.5%)	4 (57.1%)	2 (66.7%)	6 (54.5%)	18 (58.1%)
Montana/Wyoming	1 (50%)	3 (37.5%)	3 (42.95%)	1 (33.3%)	5 (45.5%)	13 (41.9%)
Personal history of cancer (*n* = 29)^a^						
Yes	Not collected	–	–	–	2 (27.3%)	2 (6.9%)
No	Not collected	8 (100%)	7 (100%)	3 (100%)	9 (72.7%)	27 (93.1%)

^a^ From risk assessment. Cancers included: breast, colon (large intestine), endometrial (uterine), kidney (renal) or urinary tract, melanoma, ovarian, pancreatic, prostate, skin cancer that is not melanoma, small intestine, stomach (gastric).

### 3.2 Barriers and facilitators along the genetic testing process

We crafted seven themes around early and late barriers to GT, early and late facilitators to GT, and testing permanence (Table 2).

**TABLE 2:** Identified themes.

Themes	
Early engagement facilitator	Family cancer history (whether significant or missing) and general curiosity were early motivators to participation
Early barrier	Technology use may lose interested individuals who also face compounded systemic barriers
Early barrier	An unknown family cancer history can exclude individuals who may benefit from testing
Late barrier	Skeptical of clinical utility
Late barrier	Privacy and insurance discrimination are top of mind
Decision permanence	Decisions to decline are not permanent
Completion facilitator	Clinical relevance and familial impact were main drivers

#### 3.2.1 Early engagement facilitators: Family cancer history (whether significant or missing) and general curiosity were early motivators to participation

I was all for the genetic testing. (Late‐2)

Participants dropped out of the testing process for a variety of reasons, but perceptions of GT were consistently positive, as was initial interest in GT. Participants expressed that the potential of GT for cancer risk was an incentive to engage with the study, regardless of whether or not they ultimately completed GT. This was primarily motivated out of concern for existing family history, “I was interested because obviously cancer runs in my family, and so there is that curiosity, am I at a higher risk? and so forth.” (Late‐4).

Other motivators included taking advantage of a free opportunity, a general desire for information, helping with research, and the opportunity to fill in missing family history. Filling in gaps was a motivator both for someone who completed testing, for filling in gaps on one side of their family, and for someone ineligible for testing: “I think, for someone in my position [adopted], genetic testing would be about the most valuable tool available in terms of assessing cancer risks.” (Early‐1).

Ultimately, barriers outweighed motivators for those who declined testing. Of the 10 test‐eligible individuals who declined, half expressed significant interest in GT, citing general curiosity or its value for their health and their families' health. Four stated that the potential for testing was an incentive for completing the risk assessment: “yeah, I was hoping that I'd get picked to get testing” (Late‐6), and four believed testing could help with cancer prevention or have direct clinical relevance. These individuals later cited concerns that outweighed their desire for testing: “I'm bummed because I really wanted to get tested. I see the drawbacks outweighing the benefits, especially since I can just assume I have a genetic marker that predisposes me to cancer and just ask for a more aggressive schedule of screenings, at least specifically for colon cancer.” (Late‐4).

#### 3.2.2 Early barrier: Technology use may lose interested individuals who also face compounded systemic barriers

I don't have a computer. I really wouldn't be able to afford one, I'm living hand‐to‐mouth now. (Early‐10)

Both individuals who did not complete the cancer history risk assessment belonged to clinics randomized to the online study arm. These individuals reported that they did not have access to, or comfort with, computers and could not complete the risk assessment as a result. However, they were eager to participate and indicated that they were information seekers. One participant highlighted the potential for this information to allow them to best prepare for the future, saying, “knowledge is power…it's power enough for me to get my affairs in order. If it's bad, I don't want to leave strings behind. I'd rather know and take care of everything.” (Early‐9). Both individuals also indicated a history of cancer; one mentioned a personal history of cancer, and the other stated, “everybody on my mother's side died of cancer.” (Early‐9). Whether these cancers were one of the assessed hereditary cancers was not ascertained, and they are therefore not included in the demographics table.

While these participants reported that technological barriers kept them from participating in the study, they also highlighted other barriers that may have become prohibitive at later stages under the traditional testing model. These included test cost, unmet access needs, and difficulty navigating the healthcare system. For example, one said:

There has always been specific things that I cannot do, like go [to the doctor] in the afternoon. Either it's too hot, or I'm too sore and tired because I start off good in the morning when I get up and progresses worse through the day with pain, movement. And because of the economic thing, I couldn't get in anyway. I couldn't even get in to see this doctor. [The cost of] gas is so high. I only have so much money. (Early‐9)

#### 3.2.3 Early barrier: An unknown family cancer history can exclude individuals who may benefit from testing

I'm adopted, so I don't really have a family history. (Early‐1)

Participants reported that the risk assessment was relatively easy to complete and similar to family history risk assessments they had completed in the past. However, of the eight ineligible individuals, three indicated that missing family history, due to adoption or lack of contact with biological relatives, hindered their ability to fill out the risk assessment accurately. One stated, “It's not like I can suddenly call my mom… like, ‘Hey, we haven't talked in years, but can you tell me your medical history?’” (Early‐6).

Missing family history reduced participants' confidence in the risk assessment's results: “I definitely took the results of this one with a grain of salt, just because the quality of the information it was giving back was probably not particularly helpful in my case.” (Early‐1) While these individuals knew they did not have a complete family history, the prospect of free GT and the possibility of filling in some missing history motivated them to attempt the risk assessment regardless.

While individuals who were missing their entire family history were the only ones completely excluded from testing, they were not the only ones to cite missing family histories. Two individuals who were eligible for genetic testing and ultimately completed it also indicated missing family history. One was eligible due to a personal history of cancer, and the other because of a known history on one side of their family.

#### 3.2.4 Late barrier: Skeptical of clinical utility

How would I benefit from genetic testing? I'd have more information, but it wouldn't impact me directly. I don't think. (Switched‐3)

Test‐eligible participants who declined testing voiced opinions that cancer could not be stopped, the results of GT could already be assumed based on cancer history, or that risk information was no longer useful to them at their age, “I am almost 60 years old, I think your perspective changes when you get older. I'm worried about other people, my kids, but let's face it, everybody's going to die of something…” (Late‐2).

The individuals who held these beliefs were generally uncertain about what GT would achieve. Five individuals noted that they were unsure about what purpose GT would serve in their lives. One participant assumed lifestyle interventions would be the main recommendation for positive results and believed that they did not need the results to implement these changes:

We should all be eating healthy, we should all be living healthy, we should all be doing these things. We all know that there's a risk, whatever level it is, that exists for us as humans, so we should be doing these things to mitigate those potential risks. If you were told that they didn't have a risk for cancer, would you go out and start doing crazier things? Probably not. So, for me I go, yeah, it would be a piece of trivia, I guess, about my life, but it would pretty much stop there. Chances of it changing much of what I do would be pretty minimal. (Late‐1)

The perception that there was limited clinical relevance to testing also influenced how stressful individuals believed the results would be. One individual recognized the importance of information but felt that while navigating other health issues, the potential for stress was too significant without the guarantee of potential interventions:

I thought, ‘I don't want to be an ostrich that buries my head in the sand. I don't want to be the see no evil, hear no evil.’ But I kind of do, because I deal with enough stress in my life right now, that if I had to worry that if I potentially had any of these, then I possibly would then be dreading ahead because maybe there were things that I couldn't do anything about, because not every one of them can you do kind of things to make sure it doesn't happen to you. I felt it was better to not know. (Late‐3)

#### 3.2.5 Late barrier: Privacy and insurance discrimination are top of mind

Everything's hackable, and then that's data that gets out there. (Late‐1)

Participants shared privacy concerns and expressed unease about who would own their genetic data, how it would be protected, or whether insurance could access their results. This worry extended into the concern that life insurance coverage could be denied in the future. For one individual, this possibility was “actually almost scarier than having cancer.” (Late‐4) Despite an interest in GT and acknowledgment of the benefits of testing, concerns about privacy and insurance discrimination were the primary reasons for not moving forward with testing among those who were eligible but declined: “only one reason [for not doing testing], and that was because it could be used against me by insurance.” (Late‐6).

Further, one participant contemplated what healthcare protections mean in the political climate at the time of interviews, stating, “I'm very, very familiar with protected health information and closed and open data sets. I get very worried, especially with the political climate we have now…who would've thought that Roe v. Wade would've been overturned?” (Late‐2), which speaks to decreasing confidence in the permanence of legal protections, “and the insurance lobby is a very, very powerful lobby. And so it just makes me very, very nervous to have anything on my medical record.” (Late‐2).

#### 3.2.6 Decision permanence: Decisions to decline are not permanent

When you asked if I'd reconsider, I think I immediately said, ‘Yes,’ almost no time. (Switched‐3)

Only two individuals of the ten who declined testing reported that, if given the opportunity for GT again, they would make the same decision, citing psychological implications as their primary concern. When the remaining eight participants were asked if they would refuse testing again, they answered that they would change their decision or gave non‐definitive responses such as “probably” and “I don't know.” This non‐committal feeling toward testing is best captured by an individual who declined testing and compared the decision to test to a pop‐up request on a computer that asks to share data with software developers—“Half of the time, I'm like, ‘Yeah, I don't care, you can have it,’ the other half of the time I'm like, ‘You don't need to know my voice recordings, no, you don't get that.’” (Late‐1). While he declined testing on this occasion, he made it clear it was not a static decision:

There are good aspects to it, and maybe someday I'll change my mind on this one. I was enough on the fence that I was willing to keep going until I got blown over by a very soft wind [a conversation with his wife]. (Late‐1)

Three individuals who had initially declined testing went so far as to request testing after being invited for the interview. One did so immediately after receiving the first email invitation, and the other two inquired about it as their interviews were concluding, “I assume based on this conversation, it's something that I can't have done at this point. Is that right? It's kind of done, the study?” (Switched‐2). All three individuals were allowed to proceed with the free testing and ultimately completed all required steps.

Of those who switched, feelings about GT were similar to those who declined and gave non‐committal responses to whether or not they would do so in the future. Two held positive feelings about GT that were initially outweighed by logistical issues and concerns over privacy and insurance implications. The third had no concerns about testing but did not see the clinical relevance.

All three participants had different catalysts leading them to move forward with testing.

The first individual initially declined testing because she was applying for life insurance and was nervous about how GT results might impact that process. After completing that process, she was eager to move forward with testing and hopeful that she could still access it through the study.

The second participant declined testing because the offer essentially fell through the cracks and said that in the future, they would “make a better effort at actually mak[ing] things like [genetic testing] happen.” (Switched‐1). During the interview, they highlighted the challenge of information overload and being inundated with scams via text, email, and phone, stating that they struggled to discern what was important. They also expressed that even with a clear understanding of the process, the amount of energy and time they had available would still be a major consideration for whether to complete testing. At the time of the interview, they were motivated to complete testing and had the time to do so. Further, the interview served as a reminder and provided the opportunity to ask questions and clarify the testing process.

The last individual did not feel strongly about their initial refusal or GT as a whole, so when they were recontacted, they decided to move forward with it. While the reasons for initially declining testing and then later completing it varied for all three individuals, the recontact was sufficient to facilitate reintroduction into the testing process.

#### 3.2.7 Completion facilitator: Clinical relevance and familial impact were main drivers

Well, my mother and my grandmother had the same cancer that they both passed from. That was a huge factor. I'm an identical twin, so it's not even just for me; it's for my sister, too. I figure it's best to know than not know. (Completed‐10)

Patients who moved completely through the testing pipeline were predominantly motivated by a concern over their family history of cancer. These participants were largely unsurprised by their risk assessment results, with many presuming an increased cancer risk from their family history alone: “it's an ongoing joke that if the war doesn't get the men, if the men don't get shipped off to war, they're probably going to… Somebody in the family's going to die from cancer.” (Completed‐4).

When asked about their perception of GT, most participants believed that it could improve their health management through increased screenings and preventative surgical options. Even if there were no current interventions for a pathogenic mutation, one participant highlighted their belief that it could still have a clinical impact “as preventative measures become more and more available.” (Completed‐7). While the possibility of prevention was the most common association with GT, concern over family and the opportunity to receive testing at no cost were also motivating factors.

People who moved forward with testing were less likely to bring up concerns, but those who did echoed the feelings highlighted by the individuals who declined testing, namely, older age, privacy, insurance discrimination, and psychological considerations. One participant commented, “The only thing that concerned me was whether it was going to affect my health insurance later if I came up with positive genetic markers. I thought about that for about, oh, I don't know, 15 s. Otherwise, it was very straightforward.” (Completed‐3). A second participant stated, “I like privacy, but also I realize that sometimes some people have, they have medical problems and they don't know about it, and that's [genetic testing] the only way we can find out” (Completed‐8). Other participants expressed that their feelings “morphed over time,” and highlighted that when they thought of GT earlier in their life, they were concerned about cost. They also didn't “want to get bad news because I have enough bad news in my life.” (Completed‐11). However, when the study offered testing, they decided to move forward with it because they were interested in having the information and “the opportunity arose” without the cost barrier. Another individual, who was 64 years old, expressed a similar sentiment to those who declined testing due to their age but was motivated by the potential positive impact on their children, commenting, “I know it interests some of my children. I think one of my daughters did some more testing … but for me, I don't feel the need to do it.” (Completed‐2).

Two individuals who completed testing also had a personal history of an assessed hereditary cancer (prostate and endometrial) and three others indicated in their interviews that they had other cancers (lung and unnamed cancers). Primary motivations for completing GT for these individuals were among the primary reasons presented above. However, one stated they do not think they would have been as motivated to complete testing if they had not had a personal history of cancer, “up until this cancer diagnosis, I really have been super healthy, no problems.” I'd probably looked at [genetic testing] and gone, “I don't know why I would do that.” (Completed‐2). Still, they indicated their ultimate motivator was providing information for their family. An additional individual with a personal history of cancer also stated a desire for GT to help discern whether their cancer was more likely to be environmental or hereditary.

## 4 DISCUSSION

This study explored why participants dropped out at different points in the GT process. We found differences in GT barriers for those who dropped out of testing early in the process versus late in the process. Earlier barriers were largely systemic (difficulty navigating technology, missing family history), while later barriers involved limited perceived relevance and privacy/discrimination concerns. These findings, along with the identified motivators for testing, offer insight into potential provider strategies for improving testing equity and uptake, such as re‐offering GT over time and focusing messaging on potential clinical actions and family impact.

The barriers identified at the earliest stage of the testing process do not represent the breadth of early perspectives, as only two individuals detailed their experiences. However, survey data were collected from approximately 7000 individuals who declined to participate in the EDGE study. The main reasons given for choosing not to enroll were ‘not interested’ and ‘no relevant family or personal history of cancer’. These responses are in line with previous literature suggesting that individuals who decline testing early have low general interest in testing (Schlich‐Bakker et al., 2007). However, those we interviewed spoke to practical limitations leading to early drop‐out: limited access to technology and a lack of knowledge about their family cancer history. Participants were interested in testing but could not participate either because the process had an online component or required knowledge of family cancer history. Both of these barriers can exacerbate inequities in GT as they systematically exclude specific groups of individuals.

The use of technology and digital tools in medical spaces aims to reduce disparities stemming from access and availability (Webster et al., 2025). However, broad integration of these tools without interventions to ensure broad access and use may contribute to specific populations being systematically unable to access health services. A scoping review evaluating health care inequities stemming from technology integration reports rurality, being from an ethnic minority, low income, poor health, and low digital literacy as factors associated with inequitable access to digital health tools (Mistry et al., 2022; Yao et al., 2022). As reliance on digital tools expands, considerations about clinic populations and ways to ensure equity must be part of the conversation. A few proposed interventions to enhance access and use may include providing technical help or establishing partnerships between libraries and clinics (Mistry et al., 2022; Yao et al., 2022).

Those who could not demonstrate a sufficient family history cited adoption and strained family relationships, scenarios for which providers may lack training (Webster et al., 2025). The CHARM study, which was looking at ways to engage populations with inequitable access to genomic testing services, identified providing testing to individuals with limited family history knowledge as essential to ensuring equitable provision of GT (Gilmore et al., 2024). To do this, the CHARM study followed an adjusted genomic testing eligibility model, which included both individuals with a sufficient family history of cancer as well as those who indicated they did not know their cancer family history. This is a middle ground between population genetic testing and targeted genetic testing, balancing resource limitations and equity.

Those who opted out of testing later were often interested in testing, but at the time of the offer, their concerns regarding insurance discrimination, privacy, and psychological impact outweighed the perceived benefits. Many individuals who declined testing at this stage were uncertain if there were actual clinical implications of testing, so the primary perceived benefit was gaining general knowledge and information. Notably, participants did not perceive their decision as permanent: some individuals requested testing after their initial decline, and others expressed the possibility of changing their mind in the future. Our findings mirror those from studies conducted in the oncological setting, despite the different implications of GT in primary care (i.e., prevention as opposed to informing treatment): both in terms of barriers to testing (Fogleman et al., 2019; Hayden et al., 2017; Keogh et al., 2017; Mallen et al., 2021; Schlich‐Bakker et al., 2007) and decision permanence (Halverson et al., 2020; Kahn et al., 2023). To add to this conversation, we were able to understand shifting motivations in real time of patients who initially opted out of testing but subsequently requested and completed it. These participants held concerns around GT similar to those who declined but changed their minds following a relatively short time interval post‐initial decline.

Our findings suggest two recommendations providers can use to address both early‐ and late‐stage barriers to GT: (1) communicating clinical and familial utility and (2) re‐offering testing over time. Those who completed testing cited the knowledge of clinical follow‐up after receipt of results as a motivator for moving forward. This is contrasted with the individuals who declined GT, who perceived limited clinical utility. For individuals who feel results would not be particularly informative for their personal care, such as individuals who are already engaging in increased cancer screenings due to age or family history, the potential positive familial impact was a motivating factor. As such, we recommend that when PCPs are engaging in conversations with patients about genetic testing, they center their messaging around the tangible impacts of GT and the potential familial benefit, especially for those who are not personally motivated to complete GT. To reinforce the clinical importance of GT and meet other patient needs, clinicians can also re‐offer testing over time.

Patient circumstances changed over the course of our study, and after a relatively brief amount of time, a few were able to proceed with testing. As such, in addition to addressing the systemic barriers inherent in the testing process, we can also potentially improve testing equity and uptake by re‐offering GT (or the collection of family/personal cancer history) over time. When GT is offered only once, individuals who could benefit may be permanently excluded from testing due to their circumstances at the time. Individuals who were systemically excluded from testing (missing family history, technology issues, health issues, poverty) may be more likely to engage with testing if providers can present additional opportunities to enter the pipeline. While the re‐offer approach may not address larger systems‐level barriers, it does allow for changes in individual circumstances. An individual's family history of cancer can change over time, shifting both their interest in and eligibility for GT. Similarly, technology access, health issues, and financial status can all change over time. Re‐offering testing would ensure these individuals can receive testing when they are situated to do so. This is supported by the three individuals whose minds changed throughout our study; two had more bandwidth upon follow‐up, and the third had a change in life insurance status. Even those who opted not to move forward with testing indicated their decision was not set in stone, suggesting that re‐offering could have a significant impact. Ultimately, reasons for not completing genetic testing are not always related to immutable or deeply held attitudes, beliefs, or values but instead are often subject to external/environmental factors and can change over time.

These findings are consistent with earlier work that found patients who initially declined GT were open to changing their minds if their physician made the recommendation again (Halverson et al., 2020). While further studies should explore the most effective means and timing to re‐offer testing, as well as provider openness to this strategy, our findings suggest that the follow‐up offer need not involve much time or effort, as we offered no additional counseling for the patients who changed their minds and completed testing. Due to the relationships between patients and their providers, existing trust, and the opportunity to provide concrete instructions, having providers re‐offer testing is likely to have the most significant impact on uptake (Bardach & Schoenberg, 2018). However, recognizing this may not always be feasible, systems‐level strategies for re‐offer should also be considered. Research around the benefits of text and simple phone call reminders for cancer screening attendance suggests a potential starting point (Breedlove et al., 2023; Kerrison et al., 2015; Taplin et al., 2000). This strategy would be responsive to patient desires for more testing opportunities while minimizing additional provider burden. This is particularly informative for the ongoing efforts to systematize GT processes via EHRs systems, providing evidence for point‐of‐care re‐offer reminders (Penn Perelman School of Medicine, n.d.).

## 5 LIMITATIONS

While the objective of qualitative studies is not generalizability, it is important to note where certain perspectives may not have been represented, as well as relevant considerations around those that were included. The vast majority (87%) of participants were non‐Hispanic white; other demographic groups may have different perspectives that are not represented here. Further, participants effectively opted into research twice, agreeing to participate in the primary EDGE study as well as this sub‐study. This may have shaped participants' motivations and concerns, such as a desire to contribute to research or greater confidence in privacy protections; our results may not generalize to clinical (non‐research) contexts. Additionally, because the EDGE study offered testing at no cost, utilized a testing company that provided genetic counseling, and had dedicated clinical staff/research members to introduce GT to patients, we were unable to thoroughly assess the associated barriers. Finally, while interviews were verbally debriefed with the analysis team throughout the process, formal/written field notes were not taken, and participants were not asked to review transcripts or findings.

In terms of the process‐oriented lens of our study, the GT process has multiple decision points, not all of which are represented. We did not reach thematic sufficiency for the considerations pertaining to those who “did not complete risk assessment.” The re‐offer of GT to those who requested it during the interview was participant‐initiated rather than by study design, so there may be self‐selection bias. Further, we don't know how others would have responded if the offer were made to them, as the openness to testing in the future was present throughout the opt‐out group, and those who switched had similar feelings and concerns around GT as the other individuals who opted out of GT. A study designed around the re‐offer of testing could offer additional insight and generalizability about why and when people may change their minds about testing.

## 6 CONCLUSION

These findings demonstrate the fluid nature of patient barriers and considerations around genetic testing and suggest potential low‐impact provider strategies for improving access to and uptake of testing. Patients' reasons for not completing testing differed based on when they dropped out of the testing pipeline, and their readiness for cancer risk assessment or testing changed over relatively brief follow‐up times. Recognizing that genetic testing is clinically important and that individual circumstances are not static, our results highlight the value of offering testing over time to improve testing equity and uptake. While this study focused on drivers of patient drop‐out and did not consider the provider side of the equation, our finding that patients' openness to testing may change over time underscores the need to identify time‐efficient, technology‐supported approaches to support providers in making repeat offers of GT. Providers often cannot address the multitude of barriers patients may be facing at any given time, but they may be able to make genetic testing more accessible by (1) tailoring messaging around prevention and familial benefits and (2) offering patients multiple opportunities to move forward with testing.

## AUTHOR CONTRIBUTIONS

Conceptualization: SBT, EMS. Data curation: TNT, EJD. Formal analysis: TNT, EJD. Investigation: TNT, EJD, SBT. Methodology: TNT, EJD, SBT, DC. Writing – original draft: TNT, FB, EJD. Writing – review and editing: TNT, FB, EJD, SBT, CW, HH, DC, EMS, SK. Visualization: TNT. Funding acquisition: EMS, SBT. Supervision: SBT, EMS, CW.

## FUNDING INFORMATION

The research detailed here was funded by the National Cancer Institute of the National Institutes of Health (NIH), Grant Office ID: A140201, Funding Source ID: 1U01CA232795‐01A1. REDCap utilization was provided with the following support: UL1 TR002319, KL2 TR002317, and TL1 TR002318 from NCATS/NIH.

## CONFLICT OF INTEREST STATEMENT

The authors declare no commercial or financial relationships that could be construed as a potential conflict of interest.

## ETHICS STATEMENT

Human studies and informed consent: The interviews described here were part of a study approved by the University of Washington Institutional Review Board (IRB), which acted as a central IRB. Study ID: STUDY00009476. Verbal informed consent was obtained prior to recording the interview, as written consent was waived by the IRB.

Animal studies: No animal studies were carried out by the authors of this article.

## Supporting information


Appendix S1

## Data Availability

The data are not publicly available at this time due to privacy restrictions.
