# Supplementary material for: Cancer genetic testing uptake in the primary care setting: Patient perspectives on barriers and facilitators throughout the testing process
Source: J Genet Couns. 2026 Mar 26;35(2):e70195. doi: 10.1002/jgc4.70195 (PMC13019829; doi:10.1002/jgc4.70195)
Supplement: Supplementary file 1 — Appendix S1 [file JGC4-35-0-s001.docx]

**Supplemental Materials**

1. EDGE Study Website – Main Page and FAQs
2. Interview Guide for participants who were offered and did not complete genetic testing: V6 on 10/25/2021
   1. Slight variations were asked based on where people were in the process. For instance, if individuals completed testing, questions around motivations for ultimately completing testing were asked.

**EDGE Website Main Page**

## About the EDGE Study

Most cancers are due to random chance. However, 5-10% of certain cancers are due to harmful genetic changes, called pathogenic variants, which can be passed down through families. This is called hereditary cancer.

The Early Detection of GEnetic Risk (EDGE) study wants to make it easier for you to know if genetic testing would be helpful, based on your personal or family history. And if testing would be helpful, we want to make it easier for you to get that genetic testing. We hope that by making genetic testing more accessible, we will save more lives by preventing cancer in those that are at-risk.

## Eligibility

Everyone has the potential to develop cancer. The EDGE study partnered with the MultiCare Health System and Billings Clinic to screen all of the patients they see over the course of a year who are age 25 or older. Depending on the results, there may be additional screening or other steps that can help your doctor reduce your risk or catch a developing cancer early.

## Genetic Testing

We worked with a genetic testing company called Color Health Inc. If personal or family history indicated genetic testing might be helpful, our study staff walked participants through the steps. We collected a mailing address and Color Health mailed a test kit to the participant’s home with instructions on how to provide a saliva sample. Results were returned through an online account and participants had the opportunity to talk with a Color Genomics genetic counselor whether their results were positive or negative.

More information can be found on our **FAQs page.**

**EDGE Website FAQs**

What kinds of questions are in the hereditary cancer screening survey?

We will ask you questions about you and your family’s cancer history. Based on your answers, you may be asked additional questions, but the survey should take less than 10 minutes to complete.

What will happen if I am a candidate for genetic testing?

If you are found to be a candidate for genetic testing, we will ask you for your email address and telephone number. A member of our study staff will reach out to you for your mailing address and to walk you through the steps of the testing, as needed.

Will this have an impact on my health insurance?

The Genetic Information Nondiscrimination Act, passed in 2008 and ensures that you cannot be discriminated against by your **healthcare insurer** or **employer** based on the results of a genetic test. To learn more about GINA protections, please visit [http://ginahelp.org](http://ginahelp.org/).

**But please note, life insurance, long-term care insurance, and disability insurance are not covered by GINA.** A positive genetic testing result may increase the premiums you would be expected to pay for a policy. If you think you might be interested in applying for either of these types of insurance, you may want to postpone genetic testing or choose not to have testing.

What if I am not a candidate for genetic testing?

We look at a combination of factors to determine whether you might benefit from genetic testing. Even if you are not a candidate for testing, it is always a good idea to consider your personal and family history. If you have any concerns, talk to your doctor. They may suggest making changes to your cancer screening schedule.

I’m afraid of what I might learn. If the genetic testing finds a mutation, does this mean I have cancer or am going to develop cancer?

No. This kind of genetic testing is not meant to test whether you already have cancer. And a change in your genes (mutation) does not mean you will definitely develop cancer.

What can I gain from this test if it doesn’t tell me if I have cancer or am going to get it?

While most results come back negative, if a mutation associated with cancer is identified there are actions you and your doctor can take. A few examples are:

i. Earlier and more frequent screenings (example: if you have an increased risk for breast cancer, your doctor might recommend mammograms at an earlier age)

ii. Preventive measures (example: taking a particular medication may help reduce the risk of developing certain cancers)

iii. Proactive care for family members (example: since the mutations we are testing for run in families, your results can help loved ones determine whether genetic testing might be helpful for them as well)

What exactly is being tested?

The test analyzes 30 genes. Genes are pieces of DNA. Each gene contains a single set of instructions. Changes (mutations) in genes can lead to mistakes in these instructions. Mutations in the 30 genes being tested have all been linked with an increased risk of cancer, and are known to run in families. These cancers include breast, colon, skin, ovarian, pancreatic, prostate, stomach, and uterine cancers. See the figure below for the list of genes being tested and which cancers they are associated with.

**Color Hereditary Cancer: The most relevant genes for common hereditary cancers**

| **Gene** | [**Breast**](https://www.color.com/learn/cancer/breast-cancer-facts-and-causes) | [**Ovarian**](https://www.color.com/learn/cancer/ovarian-cancer-facts-and-causes) | [**Uterine**](https://www.color.com/learn/cancer/uterine-cancer-facts-and-causes) | [**Colorectal**](https://www.color.com/learn/cancer/colorectal-cancer-facts-and-causes) | [**Melanoma**](https://www.color.com/learn/cancer/melanoma-facts-and-causes) | [**Pancreatic**](https://www.color.com/learn/cancer/pancreatic-cancer-facts-and-causes) | [**Stomach**](https://www.color.com/learn/cancer/stomach-cancer-facts-and-causes) | [**Prostate***](https://www.color.com/learn/cancer/prostate-cancer-facts-and-causes) |
| --- | --- | --- | --- | --- | --- | --- | --- | --- |
| [BRCA1](https://static.getcolor.com/pdfs/gene/Color_BRCA1_gene_information.pdf) | **o** | **o** |  |  |  | **o** |  | **o** |
| [BRCA2](https://static.getcolor.com/pdfs/gene/Color_BRCA2_gene_information.pdf) | **o** | **o** |  |  | **o** | **o** |  | **o** |
| [MLH1](https://static.getcolor.com/pdfs/gene/Color_MLH1_gene_information.pdf) |  | **o** | **o** | **o** |  | **o** | **o** | **o** |
| [MSH2](https://static.getcolor.com/pdfs/gene/Color_MSH2_gene_information.pdf) |  | **o** | **o** | **o** |  | **o** | **o** | **o** |
| [MSH6](https://static.getcolor.com/pdfs/gene/Color_MSH6_gene_information.pdf) |  | **o** | **o** | **o** |  |  | **o** | **o** |
| [PMS2](https://static.getcolor.com/pdfs/gene/Color_PMS2_gene_information.pdf)* |  | **o** | **o** | **o** |  |  |  | **o** |
| [EPCAM](https://static.getcolor.com/pdfs/gene/Color_EPCAM_gene_information.pdf)* |  | **o** | **o** | **o** |  | **o** | **o** | **o** |
| [APC](https://static.getcolor.com/pdfs/gene/Color_APC_gene_information.pdf) |  |  |  | **o** |  | **o** | **o** |  |
| [MUTYH](https://static.getcolor.com/pdfs/gene/Color_MUTYH_gene_information.pdf) |  |  |  | **o** |  |  |  |  |
| [MITF](https://static.getcolor.com/pdfs/gene/Color_MITF_gene_information.pdf)* |  |  |  |  | **o** |  |  |  |
| [BAP1](https://static.getcolor.com/pdfs/gene/Color_BAP1_gene_information.pdf) |  |  |  |  | **o** |  |  |  |
| [CDKN2A](https://static.getcolor.com/pdfs/gene/Color_CDKN2A_gene_information.pdf) |  |  |  |  | **o** | **o** |  |  |
| [CDK4](https://static.getcolor.com/pdfs/gene/Color_CDK4_gene_information.pdf)* |  |  |  |  | **o** |  |  |  |
| [TP53](https://static.getcolor.com/pdfs/gene/Color_TP53_gene_information.pdf) | **o** | **o** | **o** | **o** | **o** | **o** | **o** | **o** |
| [PTEN](https://static.getcolor.com/pdfs/gene/Color_PTEN_gene_information.pdf) | **o** |  | **o** | **o** | **o** |  |  |  |
| [STK11](https://static.getcolor.com/pdfs/gene/Color_STK11_gene_information.pdf) | **o** | **o** | **o** | **o** |  | **o** | **o** |  |
| [CDH1](https://static.getcolor.com/pdfs/gene/Color_CDH1_gene_information.pdf) | **o** |  |  |  |  |  | **o** |  |
| [BMPR1A](https://static.getcolor.com/pdfs/gene/Color_BMPR1A_gene_information.pdf) |  |  |  | **o** |  | **o** | **o** |  |
| [SMAD4](https://static.getcolor.com/pdfs/gene/Color_SMAD4_gene_information.pdf) |  |  |  | **o** |  | **o** | **o** |  |
| [GREM1](https://static.getcolor.com/pdfs/gene/Color_GREM1_gene_information.pdf)* |  |  |  | **o** |  |  |  |  |
| [POLD1](https://static.getcolor.com/pdfs/gene/Color_POLD1_gene_information.pdf)* |  |  |  | **o** |  |  |  |  |
| [POLE](https://static.getcolor.com/pdfs/gene/Color_POLE_gene_information.pdf)* |  |  |  | **o** |  |  |  |  |
| [PALB2](https://static.getcolor.com/pdfs/gene/Color_PALB2_gene_information.pdf) | **o** | **o** |  |  |  | **o** |  |  |
| [CHEK2](https://static.getcolor.com/pdfs/gene/Color_CHEK2_gene_information.pdf) | **o** |  |  | **o** |  |  |  | **o** |
| [ATM](https://static.getcolor.com/pdfs/gene/Color_ATM_gene_information.pdf) | **o** |  |  |  |  | **o** |  | **o** |
| [BARD1](https://static.getcolor.com/pdfs/gene/Color_BARD1_gene_information.pdf) | **o** |  |  |  |  |  |  |  |
| [BRIP1](https://static.getcolor.com/pdfs/gene/Color_BRIP1_gene_information.pdf) | **o** | **o** |  |  |  |  |  |  |
| [RAD51C](https://static.getcolor.com/pdfs/gene/Color_RAD51C_gene_information.pdf) |  | **o** |  |  |  |  |  |  |
| [RAD51D](https://static.getcolor.com/pdfs/gene/Color_RAD51D_gene_information.pdf) |  | **o** |  |  |  |  |  |  |

* CDK4: analysis is limited to chr12:g.58145429-58145431 (codon 24). EPCAM: analysis is limited to deletions that minimally encompass the 3’ end of the gene including exons 8 and/or 9. GREM1: analysis is limited to duplications that overlap the upstream regulatory region. MITF: analysis is limited to chr3:g.70014091 (including c.952G>A). PMS2: variants of uncertain significance are not reported for exons 12-15. Analysis excludes three variants commonly observed in the pseudogene PMS2CL: c.2182_2184delinsG, c.2243_2246delAGAA and deletion of exons 13-14 (chr7:g.6015768_6018727del). POLD1: analysis is limited to chr19:g.50909713 (including c.1433G>A). POLE: analysis is limited to chr12:g.133250250 (including c.1270C>G).

How will the test be paid for?

The EDGE Study will cover the cost of the testing so there are no out-of-pocket costs to you. A pre-paid code will be provided for you to set up your account and order your kit from Color Genomics.

Can you describe the testing process?

If you agree to complete testing, a testing kit will be mailed to your home. The kit will require that you provide a saliva sample and will include full instructions on how to provide the sample. It will also include a return envelope with pre-paid postage for you to ship the sample back to the Color Genomics laboratory.

You will be asked to set up an online account. In 4-5 weeks, your results will be posted to your online account and you will be notified that they are available.

If a mutation is found, a Color Genomics genetic counselor will reach out to you by phone to explain your results and to make sure you have an opportunity to ask any questions you might have. Even if no mutation is found, you may contact Color Genomics with questions at any time.

How will my genetic information be protected?

There are privacy laws in place to protect your genetic information. The Genetic Information Nondiscrimination Act was passed in 2008 and ensures that you cannot be discriminated against by your healthcare insurer or employer based on the results of a genetic test. To learn more about GINA protections, please visit [http://ginahelp.org](http://ginahelp.org/).

If you would like to know more about Color, the testing service we are using for this study, and their privacy policy, please visit [www.color.com/privacy-policy-2](https://www.color.com/privacy-policy-2.).

**Please note, life insurance, long-term care insurance, and disability insurance are not covered by GINA.** A positive genetic testing result may increase the premiums you would be expected to pay for a policy. If you think you might be interested in applying for either of these types of insurance, you may want to postpone genetic testing or choose not to have testing.

Where can I go for more information?

For more information about cancer and genetics, visit:

The American Cancer Society:
<https://www.cancer.org/cancer/cancer-causes/genetics.html>.
The National Cancer Institute:
<https://www.cancer.gov/about-cancer/causes-prevention/genetics>.
FORCE (Facing Hereditary Cancer EMPOWERED):
[https://facingourrisk.org](https://facingourrisk.org/).

For more information about the genetic testing provided by Color Genomics, visit:

[www.color.com/learn/can-cancer-be-inherited-learn-about-hereditary-cancer](http://www.color.com/learn/can-cancer-be-inherited-learn-about-hereditary-cancer).

To contact a member of the study staff, send an email to [edgestudy@uw.edu](mailto:edgestudy@uw.edu).

**EDGE Interview Guide**

**Participants who are eligible for gx testing but DID NOT complete**

***Before the call:***

1. *Send reminder 1-2 days before via email or vm.*
2. *Test audio/video setup.*
3. *Have study ID in hand.*

***Before you start recording:***

1. *Confirm availability for the scheduled duration.*
2. *Verbal consent:*
   1. *Participation is voluntary; can stop at any time; can decline any question*
   2. *Invite and answer any questions*
3. *Confirm ok to record.*

**INTRODUCTION**

Thank you for talking with me today.

Before we get started, I need to quickly double-check:

- That you are 18 or older
- That you’re a patient at the [CLINIC NAME]

Thank you.

We’re interviewing participants in the EDGE Study to learn about their thoughts and feelings about preventive cancer screening and cancer testing. Our ultimate goal is to use this information to improve the process for future patients.

There are no right or wrong answers to any of these questions. We really want to hear from people who have been through this experience, what it was like for them.

Also, just to be clear: I don’t have access to any of your medical information.

Do you have any questions before we get started?

**SECTION 1: RECEIVING RESULTS OF FAM HX RISK ASSESSMENT**

Our study records show that the family history questionnaire you completed indicated a higher than average risk of developing cancer. Different people have different reactions to receiving this kind of information.

1. **How was it for you to receive those results?**

***Probe, if needed:***

- Were the results surprising or unexpected? Why or why not?

1. **What questions did you have?**

***Probes, if needed:***

- Were they answered to your satisfaction?
- Where did you go for more information?

1. **How long did it take for you to complete the survey?**

**SECTION 2: BEING OFFERED GENETIC TESTING**

1. **What are your thoughts about genetic testing, in general?**

***Probes, if needed:***

- Good / bad
- Helpful / not helpful
- Cool, cutting edge **/** scary, risky

In the process of signing up for this study, we explained that you might be offered genetic testing, depending on your family history.

1. **How did that affect your decision to join the study?**
2. **When you were offered genetic testing, how did you feel about it?**
3. **What questions did you have?**

***Probes, if needed:***

- Were they answered to your satisfaction?
- Where did you go for more information?

1. **In your understanding, what was the purpose of genetic testing in this situation?**

***Probe, if needed:***

- What made you think so?

1. **What did you think of the way you were invited to have genetic testing?**

***Probes, if needed:***

- Did you think everyone was being asked to do so, or did you think you were getting a special invitation? If the latter, what did you think was the reason?
- Did you think it was for a research study, or part of your regular medical care?

1. **Did you think your doctor wanted or expected you to have genetic testing?**

***Probe, if needed:***

- What made you think so?

**SECTION 3: REASONS FOR NOT COMPLETING GENETIC TESTING**

We are following up with study participants like you who were eligible for genetic testing but decided not to complete the testing process.

1. **Could you tell me about why you didn’t go ahead with that?**

Sometimes people decline testing because they just didn’t have time that day, or the doorbell rang – something came up. For some people, the testing process – setting up an account, doing the test at home, sending it back -- is too complicated or time consuming. And there could be other reasons – like concerns about privacy, or unanswered questions about genetics – that might make someone say no.

I’m not here to judge you or your answers. We’d really like to understand more about why people like you might choose not to pursue genetic testing.

1. **What would you say was the main reason you declined?**

***Probes, if needed:***

- Didn’t see value:
  - Don’t think it would tell me anything new
  - Doctor didn’t ask me to – if it was important, they would
- Implementation issues:
  - Didn’t like the way I was approached
  - Thought it would cost money
  - Don’t want to be in research
- Concerns:
  - Worried about getting unwanted information
  - Privacy concerns
  - Worried about potential discrimination (e.g., employment, insurance)
  - Worried about possible follow-on expenses, procedures
- Logistics:
  - Didn’t have time
  - Forgot my glasses and couldn’t read the form
  - Other

For some people, this is a pretty straightforward decision, and they don’t spend a lot of time or energy thinking about it. For others, it feels more complicated, with different issues to weigh.

1. **How was it for you?**

***Probes, if needed:***

- Hard / easy
- Complicated / simple
- Emotionally challenging / not
- Time-consuming / quick
- Confusing / straightforward

1. **What considerations were you thinking about as you made this decision?**
2. **What else was in your mind, as you were deciding?**

***Probes, if needed:***

- Family history of cancer
- Cancer worry, psychological / emotional distress
- Environmental / occupational exposures
- Concern for family members (hereditary cancer)
- Planning for the future
- Concerns about genetic testing
- Concerns about discrimination (employment, insurance, other)
- Concerns about having results in the medical record
- Cost implications of a positive result
- Whether I want to know my risk status / potential for unwanted information

1. **What might have changed your mind, or made it easier for you to proceed with testing?**
2. **Who did you talk with as you were deciding?**

***Probes, if needed:***

- Why that person / people?
- How did they respond?
- *If not offered:* probe for family communication
- *If not offered:* probe for physician communication

1. **How did the fact that this was a genetic test figure in your thinking? Did it seem different from, say, a routine blood test?**
2. **How long did it take for you to contemplate and make the decision of whether to follow through with genetic testing (minutes and hours)?**
3. **If you had it to do over, would you make the same decision? Why or why not?**
4. **Had you ever had any other kind of genetic testing?**

***Probes, if YES and if needed:***

- For a medical reason?
- To learn more about your background or your where your ancestors were from?
- You’ve had genetic testing before. What seemed different about that from doing it as part of this study?

Some people feel that information is power – even if it’s not necessarily good news. Other people feel that there are certain things about their health – like their cancer risk – that they’d just rather not know.

1. **Where would you say you fit? Why?**

In some situations, the need for medical intervention is very clear, and we expect doctors to tell us what to do. For example, if you break your arm, you don’t usually have a long conversation about whether or not to put a cast on it.

But in other situations, it’s less clear: either the balance of risks and benefits may be uncertain, or could be different for different people. In these cases, we need to balance between what the patient wants and what the doctor thinks is best.

There is strong evidence that genetic testing for people with a family history of certain kinds of cancer can provide important information for patients and their doctors. We also know that, for people whose family history suggests increased risk, cancer screening and early detection can save lives.

1. **Given that information, how should doctors talk with such patients about whether to have genetic testing to learn more about cancer risk?**

***Probes, if needed:***

- Just offer it, give information, and leave it up to the individual patient
- Ask repeatedly (in multiple visits) if patients say no – keep the conversation open
- Tell patients they should do it
- Encourage patients to do it
- Offer to help patients do it

**SECTION 4: QUESTIONS ABOUT PREVIVORSHIP & SELF-CONCEPT**

Doctors and researchers are always working on finding better ways of helping patients identify and manage cancer risk, to help them live longer and with the best possible quality of life. One of the ideas that people are talking about right now is the concept of “previvorship.”

You’ve heard of cancer survivors – people who have had cancer and been treated successfully, so that their cancer is either cured or controlled.

Previvors are individuals who have been determined to have a predisposition, or an increased risk, for cancer but who have not yet been diagnosed with the disease. This includes people who have a family history of cancer that defines them as high risk, carry a genetic variation that is known to cause cancer, or have other things going on that are known to greatly increase risk.

1. **What do you think of the term, “previvor”?**
2. **How would it feel to you if someone referred to you as a previvor?**
3. **Can you think of a better term?**

Identifying people as previvors might create the opportunity for peer support – for example, people who have a predisposition to colon cancer could connect with each other and share their knowledge, advice, and concerns.

On the other hand, some people might worry that “previvor” could become an unhelpful label – one that could do more harm than good.

1. **What do you think?**

**WRAP-UP & HOUSEKEEPING**

1. **Is there anything else you think we should know?**

Thank you very much. I have just a few more easy questions about your age and such, and where we can send your gift card.

**Participant Demographics**

| What is your age? ***(write in)*** |  |
| --- | --- |
| What is your gender? | - Male - Female - Other |
| How would you describe your race? ***(allow multiple responses)*** | - American Indian/Alaska Native - Asian - Native Hawaiian or Other Pacific Islander - Black or African American - White - More than one race |
| Are you Hispanic or Latino? | - Yes - No |

**Address for Gift Card**

Please let me know your [MAILING ADDRESS? EMAIL ADDRESS?] so that I can send your gift card.

Thank you very much for talking with me today.
